# Integrated Analysis of N1-Methyladenosine Methylation Regulators-Related lncRNAs in Hepatocellular Carcinoma

**DOI:** 10.3390/cancers15061800

**Published:** 2023-03-16

**Authors:** Danjun Song, Xi Wang, Yining Wang, Weiren Liang, Jun Luo, Jiaping Zheng, Kai Zhu

**Affiliations:** 1 Department of Interventional Therapy, The Cancer Hospital of the University of Chinese Academy of Sciences (Zhejiang Cancer Hospital), Institute of Basic Medicine and Cancer (IBMC), Chinese Academy of Sciences, Hangzhou 310022, China; 2 Department of Ultrasound, Zhongshan Hospital, Fudan University, Shanghai 200032, China; 3 Department of Liver Surgery, Key Laboratory of Carcinogenesis and Cancer Invasion of Ministry of Education, Liver Cancer Institute, Zhongshan Hospital, Fudan University, Shanghai 200032, China

**Keywords:** hepatocellular carcinoma (HCC), N1-methyladenosine (m1A), long non-coding RNAs (lncRNAs), prognostic signature, TCGA

## Abstract

**Simple Summary:**

The relationship between m1A-related lncRNAs and HCC is still unclear. In this study, five m1A-related lncRNAs (*AL031985.3*, *NRAV*, *WAC-AS1*, *AC026412.3*, and *AC099850.4*) were identified and used to develop a prognostic signature. The prognostic signature was an independent risk factor related to OS in HCC patients. Synergistic effects on patient survival were observed after combining with *TP53* or TMB. In addition, we also screened small molecules which could be potential drugs for HCC patients. Our results suggested that five m1A-related lncRNAs generated a prognostic signature that could be a promising prognostic prediction approach and therapeutic response assessment tool for HCC patients. To the best of our knowledge, this is the first study of m1A-related lncRNAs in HCC.

**Abstract:**

N1-methyladenosine (m1A) and long non-coding RNAs (lncRNAs) play significant roles in tumor progression in hepatocellular carcinoma (HCC). However, their association with HCC is still unclear. In this study, lncRNAs related to m1A were extracted from the mRNA expression matrix in The Cancer Genome Atlas (TCGA) database. Five m1A-related lncRNAs (*AL031985.3*, *NRAV*, *WAC-AS1*, *AC026412.3*, and *AC099850.4*) were identified based on lasso Cox regression and they generated a prognostic signature of HCC. The prognostic signature was identified as an independent prognosis factor in HCC patients. Moreover, the prognostic signature achieved better performance than *TP53* mutation status or tumor mutational burden (TMB) scores in the stratification of patient survival. The immune landscape indicated that most immune checkpoint genes and immune cells were distributed differently between both risk groups. A higher IC_50_ of chemotherapeutics (sorafenib, nilotinib, sunitinib, and gefitinib) was observed in the high-risk group, and a lower IC_50_ of gemcitabine in the low-risk group, suggesting the potential of the prognostic signature in chemosensitivity. In addition, fifty-five potential small molecular drugs were found based on drug sensitivity and *NRAV* expression. Together, five m1A-related lncRNAs generated a prognostic signature that could be a promising prognostic prediction approach and therapeutic response assessment tool for HCC patients.

## 1. Introduction

Hepatocellular carcinoma (HCC) is the most common hepatic cancer, accounting for about 90% of primary liver cancers. It ranks sixth in incidence and fourth in cancer-related deaths worldwide [1]. However, HCC ranks fifth in incidence and second in cancer-related deaths in China [2]. Currently, the mechanism of HCC pathogenesis is unclear. Chronic hepatic B/C virus infection, alcoholic consumption, and non-alcoholic steatohepatitis are common risk factors for HCC development [3,4,5,6]. Although there have been significant advances in surgical and comprehensive treatments, patient prognosis remains unsatisfactory [7,8]. Therefore, it is crucial to explore the detailed carcinogenesis mechanism and identify novel biomarkers for prognosis assessment and individualized treatment.

RNA methylation modifications play a significant role in regulating post-transcriptional gene expression [9]. N1-methyladenosine (m1A) methylation in non-coding RNA (tRNA and rRNA) and mRNA has been found to be one of the essential dynamic reversible modification processes [10,11]. Similar to m6A modifications, m1A methylation is regulated by methyltransferases (writers), demethylases (erasers), and binding proteins (readers). m1A methylation is formed by adding a methyl group to the adenosine N1 position by the methyltransferases TRMT6, TRMT61A, TRMT61B, and TRMT10C [12,13], and the removal process is catalyzed by the demethylases ALKBH1 and ALKBH3 [13,14]. In addition, four specific RNA-binding proteins, YTHDF1, YTHDF2, YTHDF3, and YTHDC1, are required to complete the process [13,15]. Moreover, dysregulation of m1A methylation regulators can impact biological processes, leading to abnormal pathological development, such as cell proliferation, impaired self-renewal ability, cell apoptosis and death, and carcinogenesis [11,16,17].

Long non-coding RNAs (lncRNAs) are transcripts of more than 200 nucleotides generally not capable of encoding proteins [18], and their aberrant expression is related to tumorigenesis and progression [18]. Recently, m1A methylation regulators-related lncRNAs (m1A-related lncRNAs) were significantly correlated with cancer progression and patient survival [19,20]. However, the relationship between m1A-related lncRNAs and HCC is still unclear. Our study used The Cancer Genome Atlas (TCGA) database to perform an in-depth analysis of m1A-related lncRNAs in HCC. Additionally, characteristic clinicopathological correlation, survival analysis, predictive model construction, correlation of somatic mutations, and potential drug screening were also conducted.

## 2. Materials and Methods

### 2.1. Data Collection and Processing

**Figure 1:**
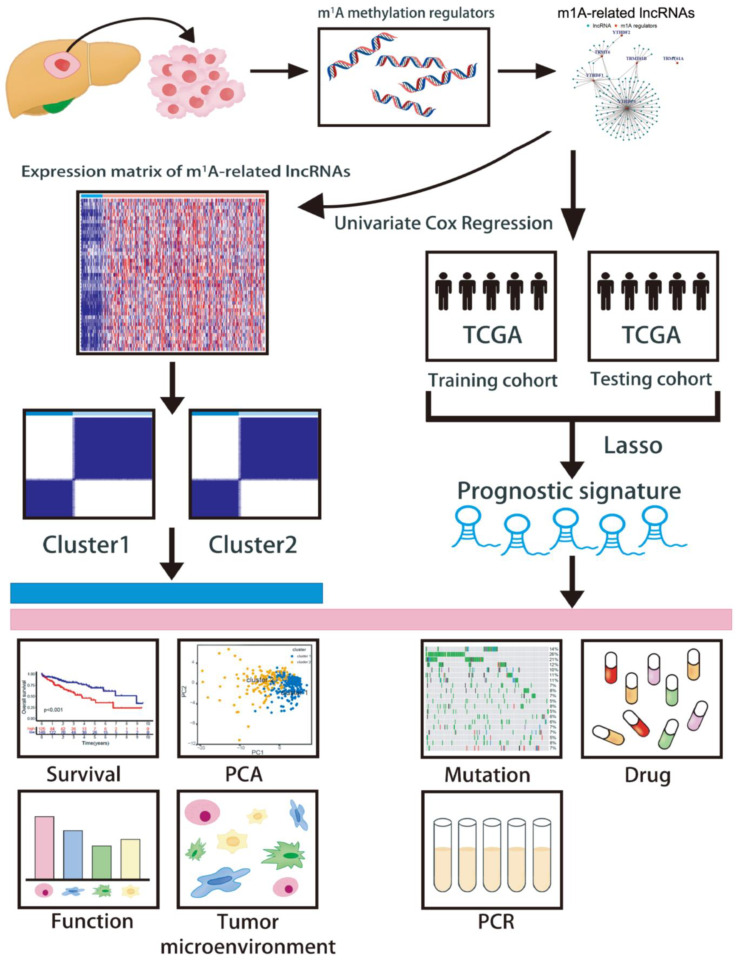
The flowchart of this study.

The flowchart of our study is shown in Figure 1. TCGA database (https://gdc.cancer.gov, accessed on 1 March 2021) was used to download the RNA-seq expression profile (FPKM values), somatic mutation data, and clinicopathological and survival information. In addition, ten m1A methylation regulators were obtained from the published literature [13,21,22], including four methyltransferases (TRMT6, TRMT61A, TRMT61B, and TRMT10C), two demethylases (ALKBH1 and ALKBH3), and four RNA-binding proteins (YTHDF1, YTHDF2, YTHDF3, and YTHDC1). The lncRNA expression matrixes and these 10 m1A methylation regulators were extracted from the mRNA expression profile using the “limma” R package, and further, 126 m1A-related lncRNAs were screened with a criterion of the absolute value of the correlation coefficient > 0.50 and *p* < 0.001. Univariate Cox regression analysis was performed with *p* < 0.05 as a cut-off value to investigate the prognosis values of 126 m1A methylation regulators-related lncRNAs. In addition, the Pan-Cancer Atlas Hub (UCSC Xena, http://xena.ucsc.edu, accessed on 1 March 2021) was used to obtain DNA and RNA stemness scores for subsequent analysis.

### 2.2. Unsupervised Clustering Analysis of m1A-Related lncRNAs

Based on the extracted m1A-related lncRNAs, unsupervised clustering analysis was performed to classify patients through the “ConsensusClusterPlus” R package. Gene set variation analysis (GSVA) was performed using the “c2.cp.kegg.v7.4.symbols” gene set obtained from the MSigDB database to compare enriched functional differences between the two clusters. In addition, the distribution of 23 immune cells between both clusters was also identified using the R packages “GSVA” and “GSEABase”. The optimal number of clusters at which the magnitude of the cophenetic correlation coefficient starts to decrease is the k value.

### 2.3. m1AScore Construction

All patients (n = 370) were randomly divided into two cohorts, including the training (n = 186) and testing (n = 184) cohorts, to develop and validate prognostic risk models. The lasso Cox regression analysis is a method to improve prediction accuracy and interpretability of statistical models and realize variable selection and regularization. Our study used lasso regression to screen the most valuable prognostic predictors in the m1A-related lncRNAs and used it to develop an m1AScore. m1AScore = (β_lncRNA1_ × expression level of lncRNA1)  +  (β_lncRNA2_ × expression level of lncRNA2)  +  ⋯  +  (β_lncRNAn_ × expression level of lncRNAn). The prognostic signature was developed based on the median cut-off of m1AScore, and patients were allocated into high- or low-risk groups.

### 2.4. Predictive Performance of the Prognostic Signature

Clinicopathological risk factors related to overall survival (OS) were investigated among the training, testing, and entire cohorts using univariate and multivariate analyses. In addition, subgroup analysis was used to detect the predictive ability of the prognostic signature in patients with different characteristics. A time-dependent receiver operating characteristic (ROC) curve was applied to investigate the predictive ability on 2-year survival using the “timeROC” R package.

Gene Set Enrichment Analysis (GSEA) was conducted using the gene set “c2.cp.kegg.v7.4.symbols” to determine significantly enriched functional pathways between the high- and low-risk groups, using the R packages “org.Hs.eg.db” and “clusterProfiler”. The top five enriched pathways in both groups were displayed.

### 2.5. Generating a Nomogram 

After integrating with clinicopathological parameters and m1AScore, a predictive nomogram was built to assess 1-year, 3-year, and 5-year OS using the “rms” R package. Calibration curves were used to identify the predictive accuracy of the established nomogram. In addition, ROC curve and decision curve analyses were used to compare predictive performance between the different clinical parameters and the prognostic signature.

### 2.6. Correlation between Single-Nucleotide Variants and the Prognostic Signature

The distribution of somatic variation between the high- and low-risk groups was investigated, and the top 20 driver genes with the highest mutational frequencies were displayed using the R package “maftool”. Subsequently, tumor mutational burden (TMB) was calculated by counting the total non-synonymous mutations, and the median TMB score was used as a cut-off value to differentiate high- and low-TMB patients. Prognostic differences among the high-TMB + high-risk (H-TMB + H-Risk) group, the high-TMB + low-risk (H-TMB + L-Risk) group, the low-TMB + high-risk (L-TMB + H-Risk) group, and the low-TMB + low-risk (L-TMB + L-Risk) group were analyzed. Furthermore, after combining *TP53* mutation status with risk levels, all patients were divided into four classes. Similarly, survival analysis among the *TP53*-mutation + high-risk (*TP53*-M + H-Risk), *TP53*-mutation + low-risk (*TP53*-M + L-Risk), *TP53*-wild type + high-risk (*TP53*-W + H-Risk), and *TP53*-wild type + low-risk (*TP53*-W + L-Risk) groups was performed.

### 2.7. Assessment of the Prognostic Signature in Immune Landscapes

Correlation analysis was used to investigate the interrelationship between 23 immune cells and m1AScore using Spearman’s method. In addition, the expression patterns of 29 immune checkpoint genes between both risk groups were also compared, according to the previous study [23]. 

### 2.8. Therapy Response Assessment of the Prognostic Signature

The “pRRophetic” R package was applied to investigate drug sensitivity between the various risk groups based on the half-maximal inhibitory concentration (IC_50_) [24]. In addition, HCC patients’ immunophenoscores (IPS) were obtained from The Cancer Immunome Database (TCIA, https://tcia.at/home, accessed on 2 March 2021) and compared between the two risk groups. IPS is a representative gene score related to immunogenicity, comprised of four determinants (MHC molecules, immunomodulators, effector cells, and suppressor cells) [25]; higher IPS indicates improved immunogenicity. The correlation between drug sensitivity and identified m1A-related lncRNAs was analyzed using the CellMiner database (version 2021.2, database 2.7) (https://discover.nci.nih.gov/cellminer/, accessed on 2 March 2021) [26] and Pearson’s correlation coefficient analysis. All drugs used for correlation analysis were approved by the Food and Drug Administration or identified by clinical trials.

### 2.9. Specimen Collection

Ten pairs of fresh tumor tissues and their adjacent paratumor tissues from ten surgically resected HCC patients were obtained from Zhongshan Hospital, Fudan University, and stored at –80 °C until RNA extraction. Informed consent forms were collected from all patients. The Ethics Committee of the Zhongshan Hospital, Fudan University, approved the protocol of this study.

### 2.10. Quantitative Real-Time Polymerase Chain Reaction (qRT-PCR)

Total RNA was extracted using Trizol reagent (Invitrogen) and reverse transcribed to cDNA using PrimeScript RT Reagent Kit (Takara). SYBR Premix Ex Taq (Takara) was used to perform qRT-PCR according to the manufacturer’s instructions. Our primers were designed with reference to the previous study [27]. We import the sequence into Primer3 for primer design. The primer sequences used in this study are listed in Supplementary Table S1. *GAPDH* was the endogenous control.

### 2.11. Statistical Analysis

The *t*-test was used to compare differences among continuous variables, and the Chi-square test or Fisher’s exact test was applied to compare differences in categorical variables. The log-rank test and Cox regression analysis were used to detect the prognostic value of these variables, while PCA was used to identify the outstanding performance of clustering information and prognostic signature. The correlation between the m1AScore and the stemness index of the tissue samples containing DNA methylation-based stemness scores (DNAss) and mRNA expression-based stemness scores (RNAss) was analyzed by the Spearman’s correlation test. All data were analyzed using R (version 4.1.1), and a two-tailed *p*-value < 0.05 indicated a significant difference.

## 3. Results

### 3.1. Identification of Two Clusters Based on the Co-Expressed lncRNAs

The expression matrixes of ten m1A methylation regulators and all lncRNAs were obtained from the mRNA profile. On correlation analysis, 126 co-expressed lncRNAs were identified as m1A-related lncRNAs. Forty-three co-expressed lncRNAs were classified as prognosis-related genes using univariate Cox regression analysis (Supplementary Table S2). The network-linked lncRNAs and m1A methylation regulators are displayed in Supplementary Figure S1A. Most co-expressed lncRNAs were related to *YTHDC1* expression. The expression patterns of these prognosis-related lncRNAs were significantly different between the normal and tumor tissues (Supplementary Figure S1B).

**Figure 2:**
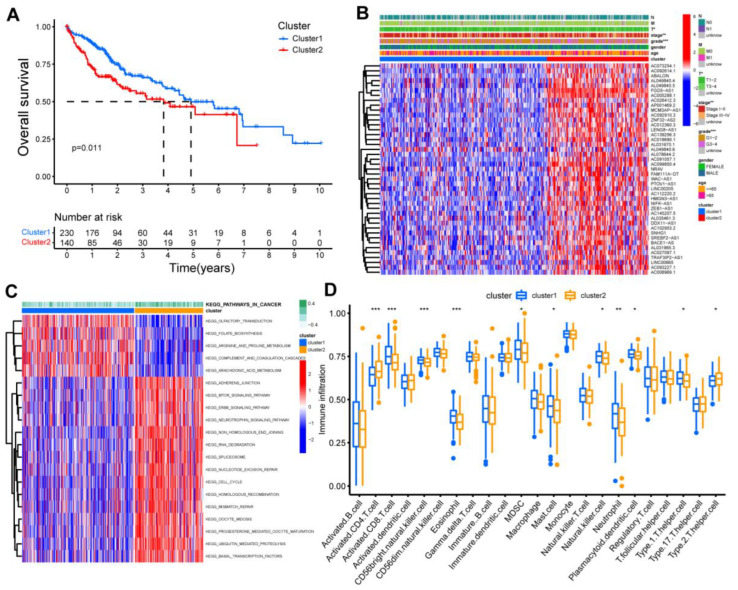
Clinical prognostic values and functions of two clusters based on the m1A-related lncRNAs. (**A**) Kaplan–Meier curve of overall survival between cluster 1 and cluster 2. (**B**) Expression pattern and clinical characteristics of cluster 1 and 2. (**C**) Identification of key pathways by GSVA analysis in both clusters. (**D**) Differential expression levels of 23 immune cells between cluster 1 and 2. *, *p* < 0.05; **, *p* < 0.01; ***, *p* < 0.001. Gene set enrichment analysis, GSEA.

In all tumor samples, two clusters were identified through unsupervised clustering analysis based on the expression matrix of screened lncRNAs (Supplementary Figure S1C). Patients were distinguished visually through PCA analysis (Supplementary Figure S1D). Survival analysis demonstrated that patients in cluster 2 suffered a worse OS (Figure 2A). A heatmap containing clinical parameters, cluster types, and lncRNA expression data was displayed to compare patient characteristics (Figure 2B). There were significant differences in T stage and pathological grade between cluster 1 and cluster 2. GSVA analysis indicated that pathways such as RNA_DEGRADATION, SPLICEOSOME, NUCLEOTIDE_EXCISION_REPAIR, and CELL_CYCLE, were enriched in cluster 2; while OLFACTORY_TRANSDUCTION, FOLATE_BIOSYNTHESIS, ARGININE_AND_PROLINE_METABOLISM, and ARACHIDONIC_ACID_METABOLISM were enriched in cluster 1 (Figure 2C). The immune cell infiltrations were compared between both clusters, and the infiltration levels of activated CD4 T cells and type2 T helper cells were higher in cluster 2 than cluster 1. In contrast, activated CD8 T cells, CD56+ natural killer cells, eosinophils, MDSCs, mast cells, natural killer cells, neutrophils, plasmacytoid dendritic cells, and type1 T helper cells were higher in cluster 1 than cluster 2 (Figure 2D).

### 3.2. Generating an m1AScore for Prognostic Prediction

A total of 370 HCC patients were allocated randomly to the training (n = 186) and testing (n = 184) cohorts, and no statistical differences were found between them (Supplementary Table S3). Furthermore, based on the forty-three co-expressed m1A-related lncRNAs, five m1A-related lncRNAs were screened as the most valuable predictors for prognostic assessment to generate an m1AScore using lasso Cox regression (Supplementary Figure S1E,F); the correlation coefficients are shown in Table 1.

**Figure 3:**
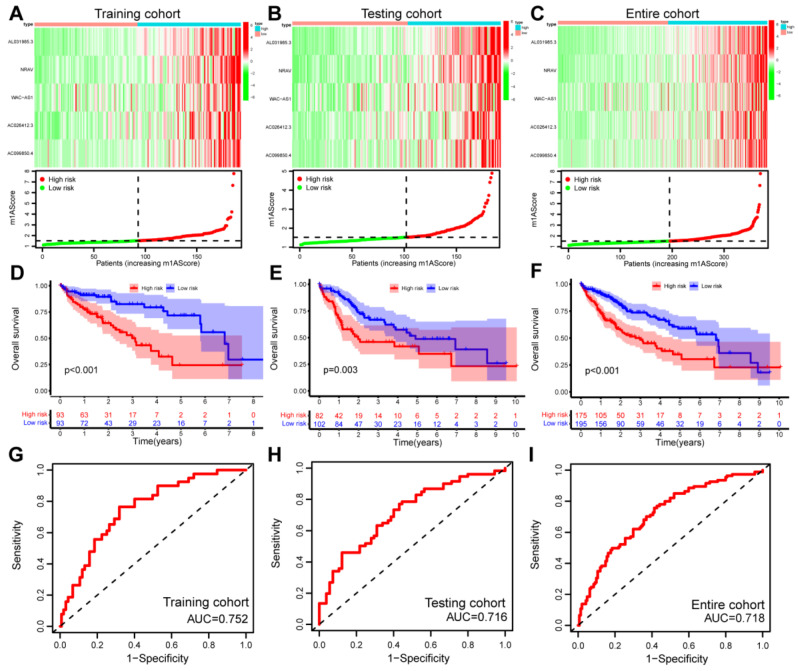
The prognostic values of the m1AScore. (**A**–**C**) Distribution of m1A-related lncRNAs and m1AScore between the high- and low-risk groups in (**A**) the training cohort, (**B**) the testing cohort, and (**C**) the entire cohort. (**D**–**F**) Kaplan–Meier survival curves of the overall survival of patients in the high- or low-risk groups in (**D**) the training cohort, (**E**) the testing cohort, and (**F**) the entire cohort. (**G**–**I**) Time-dependent ROC curve analyses in 2-year OS by m1AScore in (**G**) the training cohort, (**H**) the testing cohort, and (**I**) the entire cohort. Long noncoding RNAs, lnRNAs; Receiver operating characteristic, ROC.

Figure 3A–C shows the distribution of the relative m1A-related lncRNAs expression and m1AScore among the training, testing, and entire cohorts, respectively. Patients were distinguished visually through PCA analysis based on the prognostic signature (Supplementary Figure S1G). The Kaplan–Meier curves showed that patients with low risk displayed superior OS compared with high risk in the training, testing, and entire cohorts (log-rank test: *p* < 0.001, *p* = 0.003, and *p* < 0.001, respectively) (Figure 3D–F). The univariate and multivariate regression analysis also indicated that m1AScore was an independent prognostic indicator in both the training and testing cohorts (*p* < 0.001) (Supplementary Table S4). In addition, the time-dependent ROC curve analysis showed that the area under curves (AUCs) for 2-year OS were 0.752, 0.716, and 0.718 in the training, testing, and entire cohorts, respectively (Figure 3G–I); thus, suggesting the prediction accuracy of prognostic signature. Furthermore, subgroup analysis demonstrated that the established prognostic signature was a significant risk parameter, and that it can stratify patient prognosis by different clinicopathological characteristics (Supplementary Figure S2A–H). 

### 3.3. Function Analysis and Nomogram Construction

**Figure 4:**
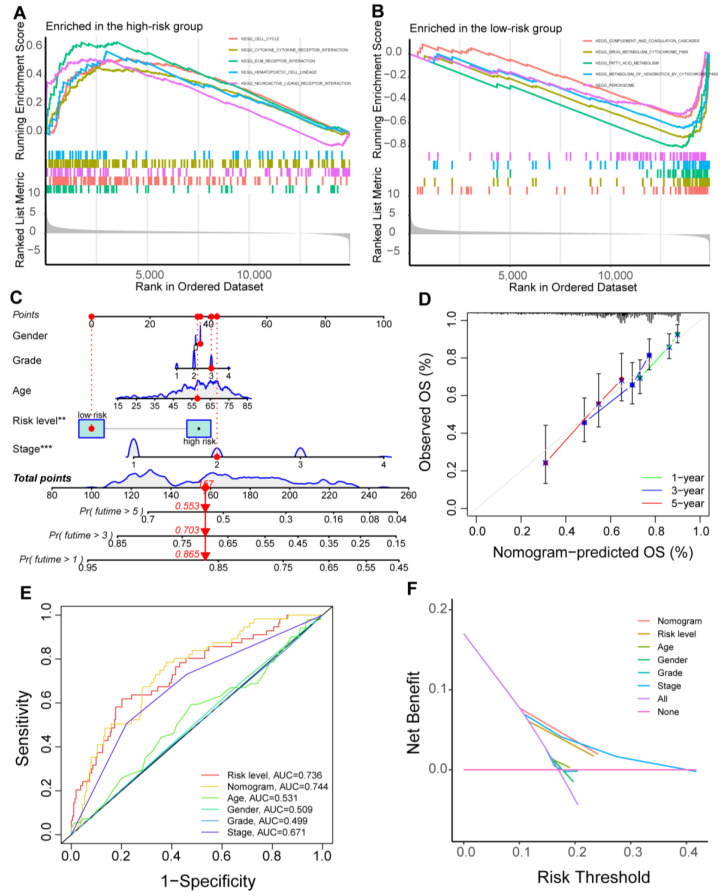
Correlation analysis between cluster types and the prognostic signature, and the construction of a nomogram. (**A**,**B**) GSEA analysis identified key pathways in high-risk and low-risk groups. (**C**) A nomogram for prediction of 1-, 3-, and 5-year overall survival in HCC patients. (**D**) The calibration curves of the nomogram predict the probability of 1-, 2-, and 3-year OS. (**E**) ROC curves of the nomogram compared with other clinical parameters. (**F**) Clinical benefit of the nomogram evaluated by decision curve analysis. Gene Set Enrichment Analysis, GSEA; Overall survival, OS; Receiver operating characteristic, ROC. **, *p* < 0.01; ***. *p* < 0.001.

A Sankey diagram was performed to analyze the interrelationship between the cluster types and the prognostic signature. It showed that most patients in cluster 1 belonged to the low-risk group with favorable outcomes (Supplementary Figure S3A), and the m1AScore in cluster 2 was higher than in cluster 1 (Supplementary Figure S3B). GSEA function analysis revealed that patients in the high-risk group were enriched in pathways such as CELL_CYCLE, while metabolism-related pathways were involved in the low-risk group patients. Thus, these findings supported the GSVA results in the above clustering analysis (Figure 4A,B).

A nomogram was established to assess the 1-, 3-, and 5-year OS (Figure 4C), and the calibration curve revealed the nomogram’s ideal consistency for predicting 1-, 3-, and 5-year survival probability (Figure 4D). The ROC and decision curves validated the nomogram’s predictive performance (Figure 4E,F). Overall, these results suggested the predictive accuracy of the generated nomogram.

### 3.4. Survival Stratification Based on the Prognostic Signature and Single-Nucleotide Variant

**Figure 5:**
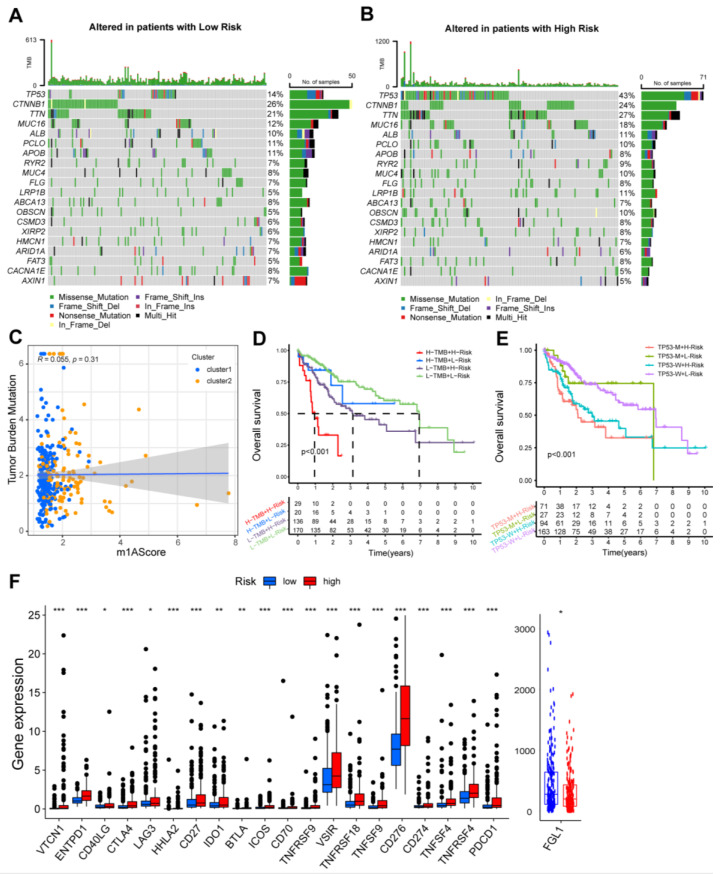
The prognostic impact of differential TMB scores or *TP53* mutation status and immune landscapes between the high-risk and low-risk groups. (**A**,**B**) The top 20 driver genes with the highest mutational frequencies between the high-risk group and low-risk group. (**C**) Correlation analysis between the TMB and m1AScore. (**D**) Kaplan–Meier curves of OS for HCC patients with different TMB scores and the prognostic signature. (**E**) Kaplan–Meier curves of OS for HCC patients with different *TP53* status and the prognostic signature. (**F**) Differential expression analysis of various immune checkpoint genes between the high-risk and low-risk groups. Tumor mutational burden, TMB; Overall survival, OS; Hepatocellular carcinoma, HCC. *, *p* < 0.05, **, *p* < 0.01; ***. *p* < 0.001.

Somatic alterations were found to affect the expression of oncogenes and tumor suppressor genes associated with tumor progression [28]. In this study, mutational frequencies of the top 20 driver genes were compared between the groups, and the results showed that the driver genes’ alteration frequencies in the high-risk group were significantly higher than the low-risk group, especially in *TP53* (43% vs. 14%) (Figure 5A,B). Because *TP53* mutation is associated with advanced tumor biological features and poor prognosis, high mutation frequencies of *TP53* and others could be the potential reasons of dismal prognosis in the high-risk group.

More and more studies have suggested that TMB could be an independent indicator for prognosis and immunotherapy [29,30]. Therefore, the combination of TMB and m1AScore could be useful for prognostic stratification and immunotherapeutic response assessment. The correlation between m1AScore and TMB was insignificant (Figure 5C). However, their combination could further stratify patient survival (Figure 5D). The OS of patients with H-TMB + H-Risk or L-TMB + H-Risk group was worse than H-TMB + L-Risk or L-TMB + L-Risk group, suggesting that prognostic signature could differentiate the prognosis in the H-TMB or L-TMB patients. These findings demonstrated synergistic effects of TMB and m1AScore in prognosis prediction.

Similarly, we further detect the synergistic effects of m1AScore and *TP53* in prognostic assessment. The prognostic signature could stratify outcomes in patients with *TP53*-M or *TP53*-W, while TMB scores failed to differentiate patient survival in high- or low-risk patients (Figure 5E), suggesting promising prognostic values of the prognostic signature.

### 3.5. The Immune Landscape of m1AScore

To explore the differences in immune landscape between the two groups, we also performed correlation analysis to investigate the interrelationship between the m1AScore and twenty-three immune cells. Activated CD4 T cells, activated dendritic cells, immature dendritic cells, plasmacytoid dendritic cells, regulatory T cells, type17 T helper cells, and type2 T helper cells were found to be positively associated with m1AScore. In contrast, activated CD8 T cells and eosinophils were negatively correlated to m1AScore (Supplementary Figure S3E). Immune checkpoint genes are closely related to the immunotherapy of malignant tumors. In this study, the expression levels of 20 immune checkpoint molecules were significantly higher in the high-risk group than the low-risk group, while *FGL1* expression was lower in the high-risk group (Figure 5F). The correlation between m1AScore and DNAss or RNAss was also investigated, and it was found that there were no strong correlations between them (Supplementary Figure S3C,D).

### 3.6. Therapeutic Response Assessment and Drug Sensitivity

**Figure 6:**
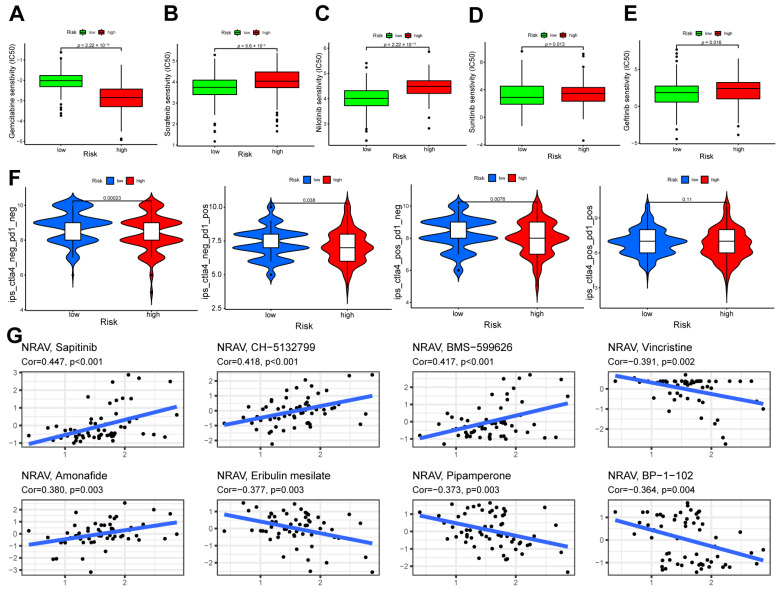
Drug sensitivity analysis and small molecule drug prediction. (**A**–**E**) IC50 sensitivity analyses of gemcitabine, sorafenib, nilotinib, sunitinib, and gefitinib between the high- and low-risk groups. (**F**) The differences of IPS-CTLA(−)-PD1(−), IPS-CTLA(−)-PD1(+), IPS-CTLA(+)-PD1(−), and IPS-CTLA(+)-PD1(+) between the high- and low-risk groups. (**G**) The scatter plot show sensitivities of small molecule drugs related with the expression of *NRAV.* (The top eight drugs are displayed).

The IC50 is a marker of response to chemotherapeutic drugs of tumor cells. The sensitivities of sorafenib, lapatinib, nilotinib, sunitinib, and gefitinib were enhanced in the high-risk group compared to the low-risk group (Figure 6A–E). In contrast, the sensitivity of gemcitabine was improved in the low-risk group, suggesting the potential therapeutic value of these drugs in various groups. Moreover, IPS-CTLA(−)-PD1(−), IPS-CTLA(−)-PD1(+), and IPS-CTLA(+)-PD1(−) showed significant differences between both groups (Figure 6F, *p* < 0.05), suggesting that the prognostic signature could be a promising predictor for assessing therapeutic drug responses.

The RNA expression data and activity of one m1A-related lncRNA, *NRAV*, were extracted from the CellMiner database. Detailed information of significantly related drugs is listed in Supplementary Table S5. The top eight drugs with the highest correlation between *NRAV* expression and drug sensitivity are displayed in Figure 6G.

### 3.7. Validation of the Expression Patterns of Five Screened lncRNAs

**Figure 7:**
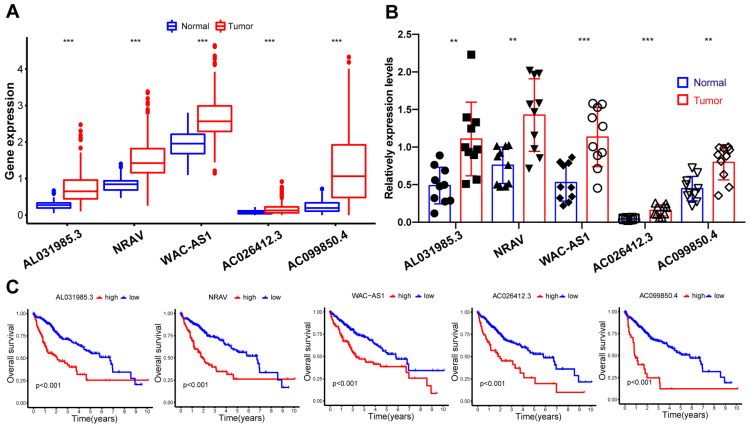
Expression patterns and prognostic values of five identified lncRNAs. (**A**) Differential expression of five lncRNAs between normal and tumor tissues in TCGA dataset. (**B**) Validation of the expression patterns of five lncRNAs based on ten cases of HCC tumor tissues and their adjacent para-tumor tissues from ten surgically resected HCC patients by qRT-PCR analysis. (**C**) The association of each lncRNA’s expression level with HCC patients’ prognosis in the TCGA database. Long non-coding RNAs, lncRNAs; Hepatocellular carcinoma, HCC; The Cancer Genome Atlas, TCGA; Quantitative real-time polymerase chain reaction, qRT-PCR. Each symbol in the figure B represents the expression level of the lncRNA in each individual. **, *p* < 0.01; ***. *p* < 0.001.

The expression patterns showed that *AL031985.3*, *NRAV*, *WAC-AS1*, *AC026412.3*, and *AC099850.4* were expressed more in the tumor tissues than normal tissues (Figure 7A). PCR also validated similar expression patterns using six tumor samples and paired normal samples (Figure 7B). The Kaplan–Meier curves demonstrated that low expression of *AL031985.3*, *NRAV*, *WAC*-*AS1*, *AC026412.3,* and *AC099850.4* is associated with better survival in the TCGA database (Figure 7C). These findings indicated the potential of these genes for diagnosis and prognostic assessment.

## 4. Discussion

Dynamic RNA methylation modifications are associated with tumor progression and regulators of m1A methylation are involved in cell apoptosis, death, and carcinogenesis [11,16,17]. A recent study demonstrated that m1A regulatory genes may play a critical role in HCC progression and could be used as biomarkers for diagnosis and prognostic assessment [13]. Previous studies indicated that RNA methylation modifications could mediate lncRNA expression [31,32], and lncRNAs affect RNA methylation modification regulators [33,34]. Wang et al. reported that the prognostic signature based on six m6A/m5C/m1A-related lncRNAs were associated with survival, immune microenvironment, TMB, and immunotherapy in head and neck squamous cell carcinoma patients [19]. However, the role of m1A methylation regulators-related lncRNAs in HCC remains unclear. To the best of our knowledge, this is the first study of m1A-related lncRNAs in HCC.

In this study, two clusters were identified based on m1A lncRNA expression, and patients in cluster 2 showed worse survival than cluster 1. An m1A-related lncRNA risk model was generated through lasso Cox regression to improve the performance of prognosis prediction. Survival analysis identified that the prognostic signature could discriminate patient outcomes among the training, testing, and entire cohorts. Univariate and multivariate Cox regression analysis demonstrated that m1AScore might be a valuable predictor for HCC patients independent of age, gender, grade, and tumor stage. A nomogram was conducted by integrating clinical parameters and the prognostic signature, and the calibration curves revealed good consistency for 1-, 3-, and 5-year survival probability. These findings thus suggested the reliable performance of established prognostic signatures in HCC patients.

The prognostic signature contained five m1A-lncRNAs, including *AL031985.3, NRAV*, *WAC-AS1*, *AC026412.3*, and *AC099850.4.* However, previous studies reported that the lncRNAs *AL031985.3, NRAV*, and *WAC-AS1* were independent prognostic risk factors in HCC and involved in the functions of pyroptosis, glycolysis, immune function, and ferroptosis [35,36,37,38]. *WAC*-*AS1* regulates *ARPP19* to promote glycolysis and tumor proliferation by sponging miR-320d in HCC [36]. These results verified the prognostic values of three lncRNAs, and there are no reports on the roles of lncRNA *AC026412.3* and *AC099850.4.* However, our study could help understand the potential function of these two lncRNAs.

HCC development and progression are associated with gene mutations [39]. Survival analysis demonstrated that the H-TMB + H-Risk group showed the worst survival and the L-TMB + L-Risk group the best prognosis, suggesting a synergistic effect after combining TMB and the prognostic signature. Moreover, the prognostic signature could discriminate OS in the H-TMB or L-TMB patients, while TMB status failed to stratify survival in the patients with low-risk; similar results were observed in the combination of *TP53* status and the prognostic signature. These findings indicated that the prognostic signature is better than TMB or *TP53* status in predicting patient OS. Moreover, combining the prognostic signature and TMB or *TP53* status can improve the prognostic values.

Exploration of response rates of chemotherapeutic drugs to tumor cells is valuable for drug screening. Drug sensitivity analysis demonstrated that sorafenib, nilotinib, sunitinib, and gefitinib had higher sensitivity in the high-risk group, while gemcitabine displayed enhanced sensitivity in the low-risk group. Sorafenib is one of the first-line therapeutic strategies for advanced HCC, and gemcitabine is an effective chemotherapeutic drug for advanced HCC in clinical practice [40,41]. Nilotinib, an orally available receptor tyrosine kinase inhibitor, can induce autophagy in HCC through AMPK activation [42]. Sunitinib is a multi-targeted receptor tyrosine kinase inhibitor, similar to sorafenib. The PRODIGE 16 study showed that TACE plus sunitinib as first-line therapy was feasible for HCC patients when surgical resection was not suitable [43]. Gefitinib, a selective EGFR tyrosine kinase inhibitor, blocks EGFR activity and has an antitumor effect on HCC development in DEN-exposed rats [44]. The sensitivity of screened lncRNAs to different small molecule drugs was also investigated. Sapitinib was highly correlated with *NRAV* expression and could induce apoptosis and suppress phospho-EGFR and its downstream pathways [45]. These findings suggested the prospect of targeting *NRAV* for HCC treatment.

Nevertheless, there are several limitations to this study. First, the m1AScore was generated based on the TCGA database, and although the testing cohort was used to verify its values, it is necessary to examine it in an independent database. Second, the expression patterns of five m1A-lncRNAs were identified by qRT-PCR; however, their function should be studied in vivo and in vitro.

## 5. Conclusions

In this study, five m1A-related lncRNAs were identified that generated prognostic signatures and can be used as an independent prognostic risk factor for HCC patients. The prognostic signature revealed its potential performance for diagnosis, prognostic prediction, and assessment of therapeutic responses.

## Figures and Tables

**Table 1:** m1A-related lncRNA risk model parameters.

Gene	AL031985.3	NRAV	WAC-AS1	AC026412.3	AC099850.4
Coef	0.2752020	0.0816187	0.0071720	0.5638878	0.0049139

## Data Availability

The data used for bioinformatic analysis that support the findings of this study are available in the TCGA database (https://gdc.cancer.gov) (accessed on 1 March 2021).

## Supplementary Materials

The following supporting information can be downloaded at https://www.mdpi.com/article/10.3390/cancers15061800/s1, Figure S1. Construction of an m1AScore. (A) 126 lncRNAs co-expressed with m1A methylation regulators. (B) Differential expression of 43 prognosis-related lncRNAs between normal and tumor tissues. (C) Consensus matrix for optimal k = 2. (D) Principal component analysis between the two clusters. (E,F) Identification of five m1A-related lncRNAs by least absolute shrinkage and selection operator (lasso) Cox regression. (G) Principal component analysis of different prognostic signatures. Figure S2. (A–H) Subgroup analysis of the prognostic signature in different populations. Figure S3. (A) The relationship among the cluster, risk score and survival state displayed by a Sankey diagram. (B) The distribution levels of m1AScore between cluster 1 and cluster 2. Correlation analysis between the m1AScore and DNAss (C) or RNAss (D). (E) Correlation analysis between the m1AScore and 23 immune cells. Table S1. The primers sequences used in the qRT-PCR experiments. Table S2. Prognostic values of co-expressed m1A-related lncRNAs. Table S3. Baseline characteristics of patients in the training, testing and entire cohorts. Table S4. Univariate and Multivariate Cox regression analysis based on different clinical characteristics and overall survival in HCC patients. Table S5. Correlation analysis between NRAV expression and drug sensitivity.
