# Supplementary material for: Integrated Analysis of N1-Methyladenosine Methylation Regulators-Related lncRNAs in Hepatocellular Carcinoma
Source: Cancers (Basel). 2023 Mar 16;15(6):1800. doi: 10.3390/cancers15061800 (PMC10046959; doi:10.3390/cancers15061800)
Supplement: Supplementary file 1 [file cancers-15-01800-s001.zip › Supplementary Tables.pdf]

Supplementary Table S1. The primers sequences used in the qRT-PCR experiments.

LncRNAs		
AL031985.3	Forward Primer	TCAGGGGTCAAAACAATCA
	Reverse Primer	TTATTTATATCAATAGTTATAGAT
NRAV	Forward Primer	TCACTACTGCCCCAGGATCAC
	Reverse Primer	TCCACTGCCAGCCCCAGGCTGG
WAC-AS1	Forward Primer	CTAACAGAACAGGAGCTC
	Reverse Primer	GTTTCCTTTAGCTCTTCTAG
AC026412.3	Forward Primer	TTTAGATATGCTAGAAGAGC
	Reverse Primer	CTGCTCCTCAGACTGTATAA
AC099850.4	Forward Primer	GGATTGCTTGAGCCTAGGAG
	Reverse Primer	TTGCGGTAGTGGTTACATGA

Supplementary Table S2. Prognostic values of co-expressed m1A-related lncRNAs

Gene	HR 95%CI	*p*-value
AP001469.2	3.440(1.291-9.164)	0.013
AL031985.3	1.879(1.547-2.283)	<0.001
SREBF2-AS1	1.458(1.170-1.818)	0.001
AC018690.1	2.104(1.435-3.085)	<0.001
LINC00665	1.120(1.040-1.206)	0.003
SNHG1	1.042(1.015-1.070)	0.002
AL049840.6	1.068(1.021-1.117)	0.004
ZNF32-AS2	1.745(1.051-2.897)	0.031
AC093227.1	1.453(1.135-1.859)	0.003
AC027097.1	2.118(1.519-2.955)	<0.001
AC092910.3	2.469(1.011-6.034)	0.047
NRAV	1.235(1.130-1.350)	<0.001
ABALON	1.918(1.086-3.386)	0.025
AL035461.3	1.080(1.010-1.156)	0.026
NIFK-AS1	1.284(1.079-1.529)	0.005
AC073254.1	5.394(1.746-16.659)	0.003
AC091057.1	2.424(1.290-4.556)	0.006
AC008969.1	2.280(1.005-5.175)	0.049
BACE1-AS	1.213(1.100-1.339)	<0.001
HMGN3-AS1	2.148(1.436-3.213)	<0.001
AC112220.2	1.573(1.097-2.255)	0.014
DDX11-AS1	4.586(2.226-9.450)	<0.001
AC139256.3	4.003(1.212-13.227)	0.023
FGD5-AS1	1.027(1.004-1.051)	0.024
WAC-AS1	1.095(1.050-1.142)	<0.001
AL031670.1	3.368(1.426-7.952)	0.006
PTOV1-AS1	1.261(1.069-1.488)	0.006
AC102953.2	1.459(1.076-1.978)	0.015
AC026412.3	14.008(4.327-45.347)	<0.001
ZEB1-AS1	1.462(1.179-1.812)	0.001
AC099850.4	1.145(1.093-1.199)	<0.001
AL049840.4	1.846(1.240-2.747)	0.003
AC005288.1	1.063(1.009-1.121)	0.022
MCM3AP-AS1	9.062(2.774-29.606)	<0.001
AC012360.3	2.453(1.325-4.541)	0.004
AL078644.2	2.619(1.212-5.660)	0.014
AC092614.1	2.771(1.184-6.482)	0.019
LINC00205	1.235(1.101-1.384)	<0.001
LENG8-AS1	1.275(1.039-1.564)	0.020
TRAF3IP2-AS1	5.904(1.697-20.538)	0.005
FAM111A-DT	1.510(1.103-2.067)	0.010
AC145207.5	1.771(1.368-2.292)	<0.001

AL049840.5	1.437(1.024-2.017)	0.036

Supplementary Table S3. Baseline characteristics of patients in the training, testing and entire cohorts

Covariates	Subgroup	Entire cohort, n (%)	Training cohort, n (%)	Testing cohort, n (%)	*p*-value
Age	≤65	232 (62.7%)	113 (60.75%)	119 (64.67%)	0.501
	>65	138 (37.3%)	73 (39.25%)	65 (35.33%)	
Gender	Female	121 (32.7%)	62 (33.33%)	59 (32.07%)	0.881
	Male	249 (67.3%)	124 (66.67%)	125 (67.93%)	
Pathological grade	Grade 1-2	232 (62.7%)	119 (63.98%)	113 (61.41%)	0.635
	Grade 3-4	133 (35.95%)	64 (34.41%)	69 (37.5%)	
	Unknow	5 (1.35%)	3 (1.61%)	2 (1.09%)	
AJCC Stage	Stage I-II	256 (69.19%)	133 (71.51%)	123 (66.85%)	0.270
	Stage III-IV	90 (24.32%)	40 (21.51%)	50 (27.17%)	
	Unknow	24 (6.49%)	13 (6.99%)	11 (5.98%)	
T stage	T stage 1-2	274 (74.05%)	144 (77.42%)	130 (70.65%)	0.141
	T stage 3-4	93 (25.14%)	40 (21.51%)	53 (28.8%)	
	Unknow	3 (0.81%)	2 (1.08%)	1 (0.54%)	
M	M0	266 (71.89%)	133 (71.51%)	133 (72.28%)	0.625
	M1	4 (1.08%)	3 (1.61%)	1 (0.54%)	
	Unknow	100 (27.03%)	50 (26.88%)	50 (27.17%)	
N	N0	252 (68.11%)	129 (69.35%)	123 (66.85%)	0.592
	N1	4 (1.08%)	1 (0.54%)	3 (1.63%)	
	Unknow	114 (30.81%)	56 (30.11%)	58 (31.52%)	

The American Joint Committee on Cancer, AJCC; Tumor-node-metastasis, TNM

Supplementary Table S4. Univariate and Multivariate Cox regression analysis based on different clinical characteristics and overall survival in HCC patients

Variables		Univariable model		Multivariable model	
		**HR (95% CI)**	***P*-value**	**HR (95% CI)**	***P*-value**
**Training cohort**					
Age		1.014(0.991-1.038)	0.230		
**Gender**	**Male and Female**	**0.522(0.295-1.922)**	**0.025**	**0.567(0.314-1.024)**	**0.060**
Grade	I, II, III and IV	1.065(0.708-1.603)	0.762		
**AJCC Stage**	**I, II, III and IV**	**1.929(1.409-2.640)**	**<0.001**	**1.775(1.279-2.464)**	**<0.001**
**m1AScores**		**1.833(1.440-2.332)**	**<0.001**	**1.652(1.272-2.146)**	**<0.001**
**Testing cohort**					
Age		1.008(0.989-1.026)	0.422		
Gender	Male and Female	1.096(0.653-1.842)	0.728		
Grade	I, II, III and IV	1.231(0.890-1.702)	0.209		
**AJCC Stage**	**I, II, III and IV**	**1.490(1.135-1.955)**	**0.004**	**1.462(1.101-1.940)**	**0.009**
**m1AScores**		**2.212(1.621-3.018)**	**<0.001**	**2.071(1.502-2.856)**	**<0.001**
**Entire cohort**					
Age		1.010(0.996-1.025)	0.174		
Gender	Male and Female	0.776(0.531-1.132)	0.188		
Grade	I, II, III and IV	1.133(0.881-1.457)	0.330		
**AJCC Stage**	**I, II, III and IV**	**1.680(1.369-2.062)**	**<0.001**	**1.579(1.273-1.957)**	**<0.001**
**m1AScores**		**1.918(1.601-2.298)**	**<0.001**	**1.735(1.433-2.102)**	**<0.001**

The American Joint Committee on Cancer, AJCC

Supplementary Table S5. Correlation analysis between *NRAV* expression and drug sensitivity

Gene	Drug	Cor	*p*-value
NRAV	Sapitinib	0.447249	0.00034
NRAV	CH-5132799	0.417782	0.000896
NRAV	BMS-599626	0.416985	0.000918
NRAV	Vincristine	-0.39145	0.001982
NRAV	Amonafide	0.380123	0.002737
NRAV	Eribulin mesilate	-0.37668	0.003013
NRAV	Pipamperone	-0.37324	0.003311
NRAV	BP-1-102	-0.3644	0.004204
NRAV	Carmustine	-0.36361	0.004295
NRAV	TAK Plk inhibitor	-0.35826	0.004944
NRAV	Dacomitinib	0.354294	0.005481
NRAV	Vandetanib	0.353646	0.005573
NRAV	Vinblastine	-0.35061	0.006025
NRAV	Afatinib	0.34442	0.007044
NRAV	Ibrutinib	0.343451	0.007217
NRAV	DMAPT	-0.34187	0.007507
NRAV	Saracatinib	0.33716	0.008431
NRAV	BMS-690514	0.336617	0.008543
NRAV	Okadaic acid	-0.33149	0.009672
NRAV	Copanlisib	0.325517	0.011151
NRAV	Lomustine	-0.32372	0.011633
NRAV	Erlotinib	0.317055	0.013576
NRAV	Denileukin Diftitox Ontak	-0.31641	0.013778
NRAV	Ifosfamide	-0.31302	0.014886
NRAV	Vinorelbine	-0.31262	0.015021
NRAV	Imexon	-0.31026	0.015841
NRAV	GSK-2126458	0.308423	0.016505
NRAV	Paclitaxel	-0.30261	0.018769
NRAV	CT-32228	-0.30201	0.019018
NRAV	Curcumin	-0.30116	0.019372
NRAV	Arsenic trioxide	-0.30016	0.0198
NRAV	Barasertib	-0.29982	0.019945
NRAV	GSK-461364	-0.2998	0.019953
NRAV	VINORELBINE	-0.29743	0.021002
NRAV	Alectinib	-0.29734	0.021044
NRAV	Gefitinib	0.295255	0.022007
NRAV	Volasertib	-0.29126	0.023955
NRAV	Everolimus	0.29048	0.024354
NRAV	Pictilisib	0.287325	0.026017
NRAV	PX-316	-0.27832	0.031296
NRAV	Sunitinib	-0.27799	0.031509
NRAV	AEG-40730	-0.27538	0.033204

NRAV	SR16157	0.274028	0.034116
NRAV	EMD-534085	-0.27361	0.034401
NRAV	XAV-939	0.272698	0.03503
NRAV	Osimertinib	0.27158	0.035815
NRAV	DOLASTATIN 10	-0.26715	0.039069
NRAV	Des-fluoro-TAK-960	-0.2671	0.039104
NRAV	Apitolisib	0.266291	0.039726
NRAV	Acalabrutinib	0.265382	0.040433
NRAV	KX-01	-0.26446	0.04116
NRAV	DACARBAZINE	-0.2621	0.043067
NRAV	Crenolanib	-0.26146	0.043599
NRAV	Irofulven	0.259353	0.045386
NRAV	5-Fluoro deoxy uridine 10mer	0.256148	0.048215
