# Supplementary figures and images for: Integrated Analysis of N1-Methyladenosine Methylation Regulators-Related lncRNAs in Hepatocellular Carcinoma
Source: Cancers (Basel). 2023 Mar 16;15(6):1800. doi: 10.3390/cancers15061800 (PMC10046959; doi:10.3390/cancers15061800)

**Figure S1**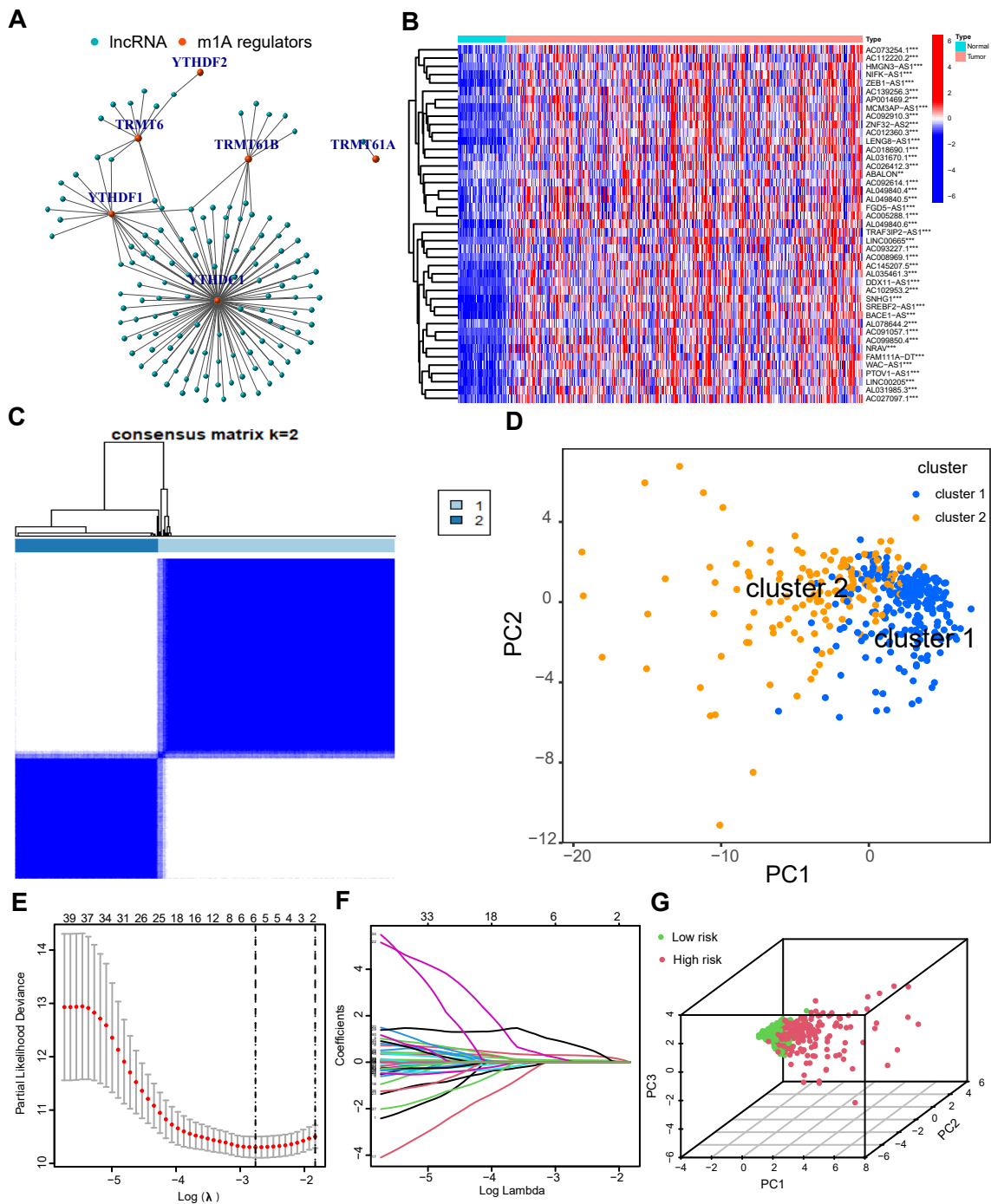

Supplement: Supplementary file 1 [file cancers-15-01800-s001.zip › Supplementary Figure S1.pdf]

**Figure S2**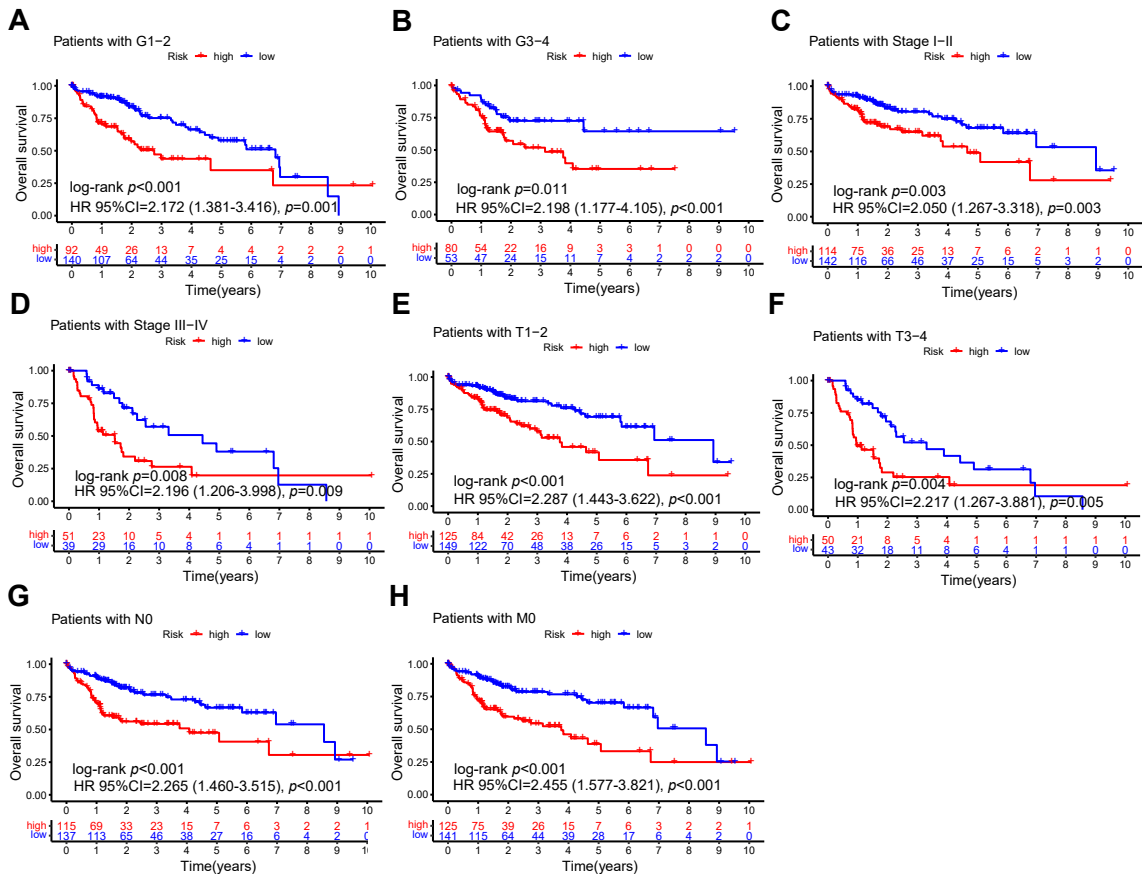

Supplement: Supplementary file 1 [file cancers-15-01800-s001.zip › Supplementary Figure S2.pdf]

**Figure S3**

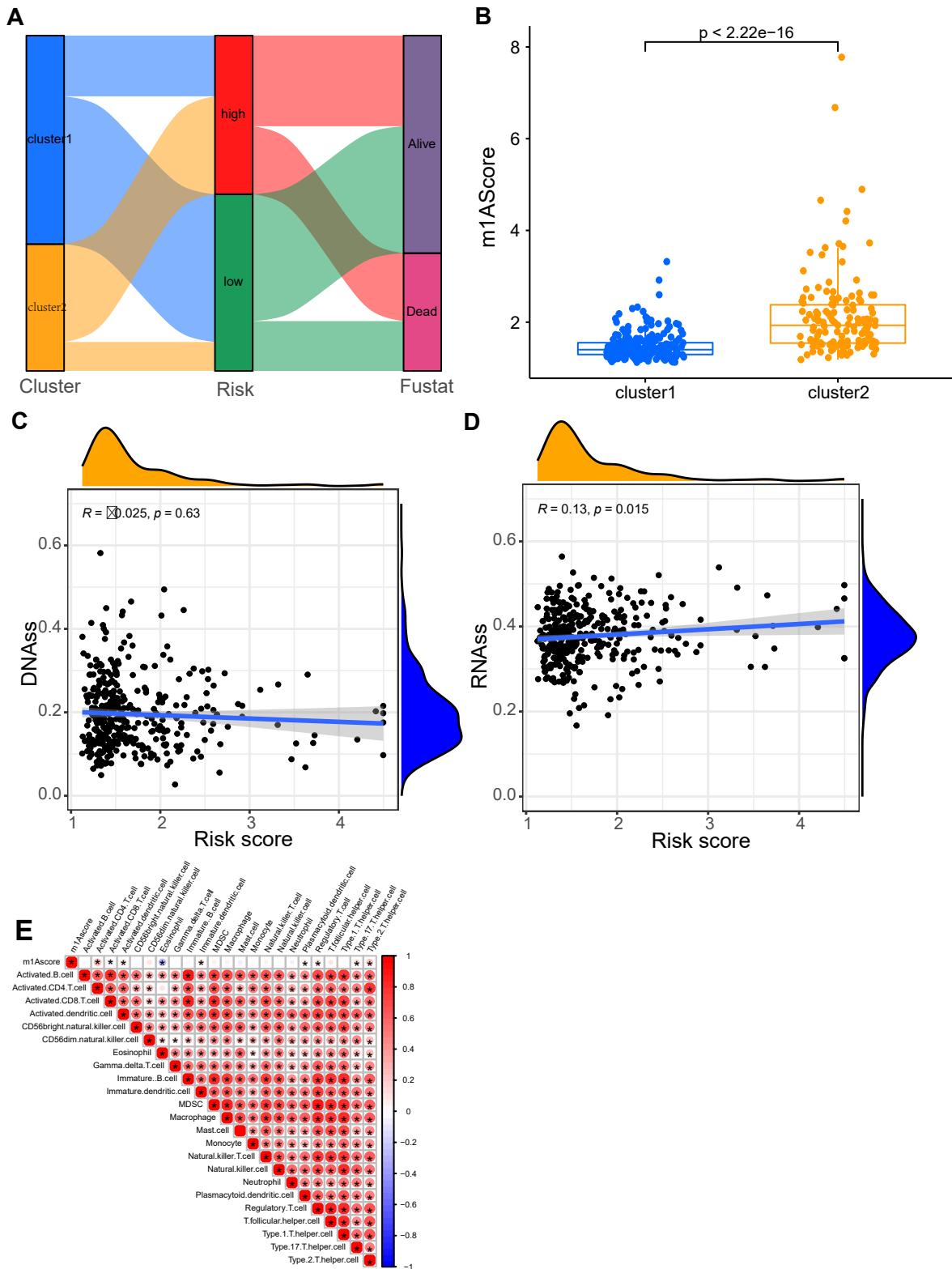

Supplement: Supplementary file 1 [file cancers-15-01800-s001.zip › Supplementary Figure S3.pdf]
